# Acupuncture in the Inpatient Acute Care Setting: A Pragmatic, Randomized Control Trial

**DOI:** 10.1155/2012/309762

**Published:** 2011-06-22

**Authors:** Jeannette Painovich, Patricia M. Herman

**Affiliations:** ^1^Cedars-Sinai Medical Center, Los Angeles, CA 90048-1865, USA; ^2^Evaluation, Research and Development Unit, University of Arizona, Tucson, AZ 85721-0462, USA

## Abstract

*Purpose*. To evaluate the acceptance and effectiveness of acupuncture in a hospital setting. *Methods*. This 18-month pragmatic randomized controlled trial used a two-tiered consent process for all patients admitted to the acute care unit by study physician groups. The primary study comparison was between those randomized (using biased-coin randomization after initial consent) to be offered acupuncture or not. The primary outcome was length of stay (LOS). Other measures include costs, self-reported anxiety, depression, health status, and patient satisfaction. *Results*. Of the 383 patients consented to the study, 253 were randomized to be offered acupuncture, and 130 were not offered acupuncture. Of those offered acupuncture, 173 (69%) accepted and received daily acupuncture. On average, patients offered acupuncture had longer LOSs (4.9 versus 4.1 days) than those not offered acupuncture (*P* = .047). Adjustment for diagnosis and severity mix reduced this difference and its significance (*P* = .108). No other significant differences in outcomes were found. Patients who were more anxious (*P* = .000) or depressed (*P* = .017) at admission tended to more often accept acupuncture when offered. *Conclusion*. Acupuncture is accepted by a majority of hospitalized acute care patients. However, it did not reduce LOS in this already short-stay population.

## 1. Introduction

According to the Health and Human Services Agency for Healthcare and Research Quality, the US spends approximately one-third of its healthcare dollars on hospital care, making hospitalizations the single most expensive component of the health care system [1]. Cost savings, accrued through the steady decline in length of stay (LOS), advances in technology and drug therapies, and managed care leveled off by 1998 and have since been replaced by an increasing rate of hospital spending [2]. These rising costs, coupled with the fact that our aging population is estimated to increase the need for acute care beds by as much as 46 percent by 2027, accelerate the need for new and innovative ways to help reduce LOS and defer the ever-growing cost of hospital medicine [3]. 

Acupuncture has been used in China for years as part of the practice of traditional Chinese medicine (TCM) and has continued to evolve over thousands of years [3]. TCM medical theory holds that health occurs when the patterned energy flow throughout the body is balanced. Acute illness occurs, and hospitalization is sometimes needed, when a major state of imbalance or disruption ensues. If the use of acupuncture can correct these imbalances, recovery is accelerated. China's current hospital system often offers the integration of TCM and western medicine. Although acupuncture is widely used and accepted in the US in outpatient settings, this approach of having both eastern and western modalities available for hospital inpatients is infrequently practiced. 

A number of studies (mostly outpatient) have been conducted to examine acupuncture's effectiveness. The use of acupuncture has been shown to be better than placebo (sham acupuncture) in the treatment of knee pain and gastroesophageal reflux [4, 5] and better than no acupuncture for patients with low back pain, headaches, and depression [6–10]. In pulmonary disease, acupuncture has been found to be safe and potentially effective in treating bronchial asthma and chronic obstructive pulmonary disease (COPD) [11]. Multiple studies have also shown that acupuncture may be cost effective for chronic headaches (including migraines), low back pain and osteoarthritis pain [12–17]. In the inpatient setting, the use of adjunctive acupuncture has been shown to reduce pain, nausea, and vomiting and decrease the need for narcotic use [18, 19]. To date, however, no randomized controlled trials have examined acupuncture's acceptance in the inpatient hospital setting or effectiveness in reducing associated costs and length of stay.

## 2. Subjects and Methods

The study was an 18-month pragmatic randomized controlled effectiveness trial of acupuncture to reduce length of stay (LOS) and costs and improve patient outcomes and satisfaction in a hospital setting. It was conducted from June 2007 through December 2008. Study design, consent forms and procedures were approved by the Western Institutional Review Board. 

### 2.1. Participants

All patients admitted into the hospital by the study's six participating physician groups, who account for a very small percentage of total admitting physicians (<5%), were eligible. Initially, the types of diagnoses targeted included acute myocardial infarction (AMI), coronary artery bypass graft (CABG), congestive heart failure (CHF), cerebral vascular accident, asthma, COPD, pneumonia, laminectomy, total hip replacement, and total knee replacement. However, at six-month lower-than-expected admission of patients into our initial diagnostic categories required an expansion of our inclusion criteria as an attempt to ensure sufficient numbers of participants for subgroup analyses. Thereafter, all patients from the study's physician groups were approached to participate. Exclusion criteria for all patients included cognitive impairment (i.e., inability to read and/or properly fill out a questionnaire). Exclusion criteria for those patients offered acupuncture included a concurrent diagnosis of sepsis or skin infection.

### 2.2. Randomization

A two-tiered consent process was used to allow separation of patient preference for acupuncture from willingness to participate in a research study [20]. All eligible patients were asked to give informed consent to provide data on their general health and well-being via a short questionnaire at admission and discharge and to allow hospital billing records to be analyzed. Patients who consented to the collection of data were then randomized to be offered acupuncture (offered) or not (not offered). Those who were randomized to be offered acupuncture and accepted (offered-accepted) were then subject to a second, separate informed consent process. Because not all patients offered acupuncture were expected to accept it, a two-thirds/one-third biased coin randomization was used to ensure sufficient numbers of patients in the offered-accepted group [21]. The biased coin design also allowed for ethical adjustment, as needed during the study, of the numbers of patients offered acupuncture to balance patient load and acupuncture staff availability. The study acupuncturists (deindentified as such) approached and enrolled all study participants.

### 2.3. Outcomes

The primary outcome was length of stay (LOS). These data as well as costs were obtained from the hospital's decision support system which utilizes data from its patient accounting system and general ledger. Several self-reported measures of health and patient satisfaction were also gathered as secondary outcomes. The hospital anxiety and depression scale (HADS) is a validated measure of anxiety and depression specifically designed for use in a nonpsychiatric, hospital setting [22, 23]. A single-item measure of general health (“In general, how would your rate your overall health?”) with a five-point response range from “excellent” to “poor,” and two items capturing patient satisfaction (“Using any number from 0 to 10, where 0 is the worst hospital possible and 10 is the best hospital possible, what number would you use to rate this hospital during your stay?” and “Would you recommend this hospital to your friends and family?”) from the hospital's regular patient survey were also used. Single-item measures of overall health status have been found to be valid in several studies [24, 25]. The self-report instruments were administered by the study acupuncturists at admission after informed consent and at discharge. The patient satisfaction questions were only administered at discharge.

### 2.4. Intervention

 All patients received usual care during their hospital visit. Those who were offered acupuncture and accepted were given daily (unless unavailable due to scheduling conflict) acupuncture treatments in addition to usual care and could deny treatments if so desired. In keeping with a pragmatic design, treatments were rendered in standard TCM practice. Each treatment and treatment style was based on individual patient presentation, diagnosis, and chief complaints. 

Acupuncture style varied according to practitioners' experience, preference, and patient presentation. Kiiko Matsumoto (Japanese hara diagnosis), Master Tung, and TCM styles were all employed with the first two used most frequently. Depth of needle insertion varied from superficial needling (Kiiko Matsumoto style) to 0.3 to 1.2 mm in depth (Master Tung and TCM styles) depending on location of needles. Number of needles inserted varied depending on patient presentation and diagnosis. Electrical stimulation (2/100 Hz with a dense/sparse mixed wave form) was used when deemed appropriate (e.g., in TCM points used for musculoskeletal complaints); otherwise, all stimulation was manual. Once needles were inserted per assessment, they were retained for approximately 20–30 minutes. Four licensed, hospital credentialed practitioners with experience ranging from six to 22 years conducted the acupuncture treatments.

### 2.5. Statistical Analysis

In order to determine the effectiveness of *offering* acupuncture to this patient population, the primary study comparison was between the patients randomized to the offered and not offered groups. The second comparison was between the offered-accepted and offered-refused groups to determine the proportion and types of patients likely to accept acupuncture if it was offered. It was not expected that acupuncture would be effective in all acute care patients. Therefore, subgroup analyses were planned for each of the major diagnostic categories and for smaller subgroups as numbers allowed.

In order to allow comparison of LOS and costs across patients with a variety of diagnosis and procedure codes, each patient's actual LOS and cost was divided by an estimate of the *expected* LOS or cost for that patient's all-patient refined diagnosis-related group (APR-DRG); creating actual-to-expected ratios for LOS and for costs. The APR-DRG codes indicate patients with similar diagnoses, procedures, and severity levels, and were determined using the 3M Health Information Systems algorithms. Expected LOS and cost values were estimated as the average LOS or cost for all patients with the same APR-DRG seen across the entire hospital inpatient population during the study period. 

Comparison of the means of continuous variables between groups utilized *t*-tests, and comparison of categorical variable counts utilized *χ* ^2^ tests. Subgroup analyses to test whether the offer of acupuncture had a differential effect by APR-DRG group were performed by creating a set of interaction terms between treatment group assignment and APR-DRG and comparing the explanatory power (measured by *R* ^2^ and using an incremental *F*-test) of an equation containing these terms to one that only included APR-DRG [26, 27]. If the inclusion of the interaction terms does not add sufficient explanatory power (i.e., the *P* value for the incremental *F*-test is > .05) then the hypothesis that the offer of acupuncture had no effect on any APR-DRG group cannot be rejected. Statistical analyses were performed using Microsoft Office Excel 2003 (Redmond, Wash, USA) and SPSS 15.0 for Windows (Chicago, Ill, USA).

## 3. Results

**Figure 1:**
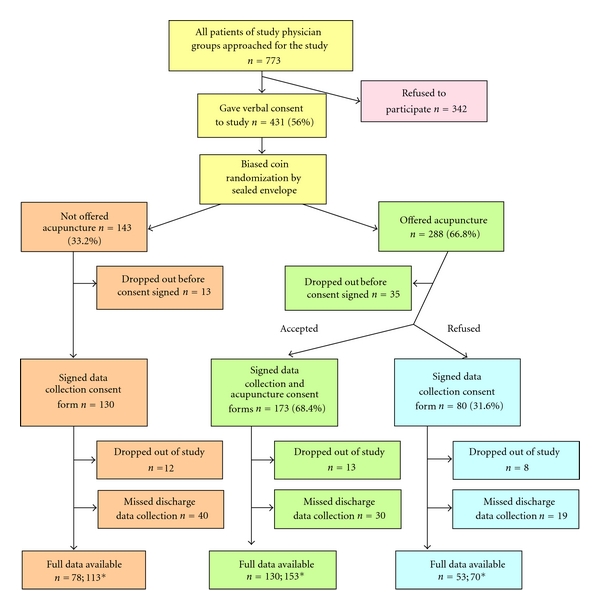
Flow of patients through the study. Diagram illustrating the two-tiered consent process and flow of acceptance and refusal of patients to both data collection and acupuncture intervention. *The first number represents self-report data; the second represents cost and LOS data, some of which were unavailable for analysis because of an insufficiency of similar patients from which to estimate expected LOS and cost values.

As can be seen from Figure 1, 773 patients were approached for the study, 56 percent gave initial verbal agreement to participate, and 50 percent (383) signed a consent form and actually participated. Patients often asked for time to read and discuss the informed consent documents with family members. Since these patients had a chance of later being randomized to be offered acupuncture and requiring a second consent, we randomized patients after verbal agreement so that both consent documents could be read and discussed. Some of these patients ultimately decided not to participate (35 in the offered acupuncture group and 13 in the not offered group). However, the rate of refusal did not differ significantly between groups (*P* = .341). 

Once patients were consented to the study, they did tend to stay. Only 9 percent (12/130) of the not offered group and 8 percent (21/253) of the offered group (8% of the offered-accepted group and 10% of the offered-refused group) dropped out of the study. Dropouts were not statistically related to age, sex, medical group, diagnosis group, treatment group, admission self-rating of overall health, admission depression, or admission anxiety. Dropping out of the study was, however, significantly related to the patient's race and ethnicity (*P* = .009). Patients who identified as Pacific Islander/other had the highest dropout rate (23%), Caucasians had a dropout rate of 9 percent, and those who identified as Hispanic, Asian, or African American had the lowest dropout rates (4%, 2%, and 2%, resp.). As noted in Figure 1, discharge self-report data was missing for a substantial portion of participants, mainly due to patients leaving the hospital before discharge data could be collected. In response to this discovery, the acupuncturists changed their daily shift from afternoon to morning, which reduced missing self-report data from about 45 percent during the first six months of the study to about 18 percent during the last 12 months. Length of stay and cost data were available on all patients who completed the study.

Table 1 shows the data at admission (including later dropouts) for each group. The comparison between the first two columns (not offered and offered acupuncture) shows the success of the randomization. Ideally, the mean values and percents in these two columns would be equal. In general, they are fairly similar and none of the differences seen are statistically significant (*P* < .05). The comparison between the last two columns indicates the types of patients who accept or refuse acupuncture when it is offered. In general, the groups are fairly similar in terms of the variables measured. However, patients at admission who were more anxious according to the HADS anxiety (*P* < .001) and who were more depressed according to the HADS depression (*P* = .017) were more likely to accept acupuncture when it was offered. 

Table 2 shows the diagnosis groups (defined by groups of APR-DRGs) for the patients for which these codes were available—that is, excluding dropouts. Patients with a wide variety of diagnoses were seen and they were fairly evenly dispersed between those offered and not offered acupuncture. Overall, 69 percent of those offered acupuncture accepted it. 

On average patients offered acupuncture had a significantly (*P* = .047) longer LOS than those not offered acupuncture—on average 0.8 (95% CI: 0.01–1.5) days longer. However, the average *expected* LOS estimates for each group based on their APR-DRG groups (4.7 days for the offered group and 4.4 days for the not offered group, data not shown) indicate that more patients who were expected to have longer LOS, given their conditions, were randomized to the group offered acupuncture. Therefore, the more appropriate comparison is between the average actual-to-expected LOS ratios for each group.  Table 3 shows the average actual-to-expected ratios for LOS and total costs across all participants and by group for each APR-DRG grouping for which there were at least 10 patients. As can be seen, across all participants the average actual-to-expected LOS ratio for those not offered acupuncture was 0.94 (i.e., on average those not offered acupuncture had LOSs that were 94% of what was expected given their APR-DRGs) compared to 1.04 for those offered acupuncture. This difference is not statistically significant (*P* = .108). In addition, no statistically significant differences were found between groups in terms of changes in anxiety, depression, general health status, and patient satisfaction.

Patient satisfaction was equally high in both groups. The average rating of the hospital on a 0-to-10 scale was 8.5 in the offered acupuncture group and 8.6 in the not offered group, and 97 and 96 percent, respectively, of each group indicated that they would recommend the hospital to friends and family.

### 3.1. Subgroup Analyses

The comparisons shown in Table 3 of actual-to-expected ratios for LOS and costs between study groups vary widely across APR-DRG groupings, and few of the differences yield *P* values less than .05. However, due to the large number of comparisons made, a more appropriate test of whether offering acupuncture has an effect on any APR-DRG group is to compare the results of two regression equations—one (the restricted model) allowing the actual-to-expected ratios for LOS and cost to vary by APR-DRG and the other (the full model) allowing these ratios to vary by APR-DRG and by a set of interaction terms between group assignment and each APR-DRG. The incremental *F* statistic for the comparison of the two models for LOS is 0.994, which with 15 and 306 degrees of freedom yields a *P* value of .461. The incremental *F* statistic for total costs is 1.566 with the same degrees of freedom for a *P* value of  .082. Therefore, there were no statistically significant differences in LOS or costs by treatment group across the APR-DRG-defined subgroups.

## 4. Discussion

To our knowledge, this is the first randomized controlled trial examining the effectiveness of acupuncture in the acute care setting for the outcomes of length of stay, costs, and acceptability. According to our findings, acupuncture had a high acceptance rate (69%) when offered to acute care patients. Treatments were well tolerated, conflicts with scheduling were rare, and no adverse events were reported. Although overall the offered acupuncture group averaged a longer LOS, after correcting for the fact that the randomization procedure allocated more longer-stay diagnoses to the group offered acupuncture, this difference was no longer significant. There were no overall group difference seen in cost of care, patient satisfaction or perceived health outcomes. Finally, patients who were more anxious and depressed at admission tended to accept acupuncture when it was offered. This finding warrants further analysis into what types of patients may be open to acupuncture intervention and why.

In retrospect, given an average acute care stay of only 4.6 days, impacting LOS and/or costs via acupuncture presented a difficult task. Over the past decade, continued efforts in the biomedical field to reduce LOS have made little impact [28]. Even the efforts of hospitalists, acute care specialists who are intimately involved in patient care from admission to discharge, have reduced LOS an average of 0.4 days and had little impact on overall costs for the diagnoses of pneumonia, heart failure, chest pain, acute exacerbation of chronic obstructive pulmonary disease, or acute myocardial infarction over a three year period [29]. In addition, some experts question how much LOS actually effects a reduction in costs. One study examining the true impact of LOS on cost found that in an LOS of four days, although the last full day represented 25% of the total LOS, it only was responsible for 2.4% of the total cost of care [30]. This statistic is not surprising given that the bulk of the cost of care is incurred during the early diagnostic and intervention phase, while the final days are essentially recuperative.

Based on the results of this particular study, implementing acupuncture in the acute care setting to reduce LOS is not supported. However, given research findings in more specific inpatient arenas, further examination of acupuncture integration seems warranted. For example, in the postoperative setting, acupuncture has been found effective in reducing pain and narcotic use [31, 32]. In addition, its use has demonstrated a significant reduction in the incidence of postoperative opioid-related adverse effects, including nausea, pruritus, dizziness, sedation, and urinary retention [33]. This are clinically relevant findings given that a previous study suggests that patients place almost equal importance in the type and severity of the side effects as they do analgesia efficacy when assessing the outcome of acute pain management [34]. One recent study also suggests that electroacupuncture inhibits the innate immune response elicited by surgical trauma, thus inhibiting trauma-induced proinflammatory cytokine expression [31, 32, 35]. 

One limitation to note is that due to the exploratory nature of the study, it included patients with a wide variety conditions, necessitating a scheme by which to “level the playing field” with regard to expected LOS and costs. Our analyses were highly dependent on APR-DRG coding to identify comparable patient subgroups. Whereas APR-DRG codes were specifically developed to group hospitalized patients by intensity of resource use, patients within single codes can still experience widely varying costs and LOS. APR-DRG codes are assigned after a patient is discharged and depend to some extent on patients' experience during their hospital stay. Therefore, if acupuncture can help patients heal faster and avoid some additional procedure or infection, it is possible that a patient, who would have otherwise been assigned one APR-DRG at discharge, was actually discharged with a lower-rated APR-DRG (e.g., one with a lower severity level) because of acupuncture treatment. If this was ever the case, our actual-to-expected cost and LOS ratio approach may have biased our results against acupuncture. In addition, having the treatment acupuncturists administer the self-report questionnaires, especially at discharge, may have biased answers due to therapeutic alliance with the provider.

## 5. Conclusion

According to this study, acupuncture is a highly acceptable adjunctive medical modality that could be integrated into current hospital systems. However, in this already short-stay population, it was unable to reduce length of stay.

## Figures and Tables

**Table 1:** Comparison of groups at admission.

	Not offered acupuncture (*n* = 122)*	Offered acupuncture (*n* = 247)*	Offered-accepted (*n* = 172)*	Offered-refused (*n* = 75)*
Age (Yrs)-mean (SD)	62.8 (16.9)	63.6 (16.2)	63.4 (15.6)	64.0 (17.4)
Female–percent (#)	54 (66)	45 (111)	45 (77)	45 (34)

Race–percent (number)				

Caucasian	28 (34)	35 (86)	32 (55)	41 (31)
Hispanic	39 (47)	33 (82)	35 (61)	28 (21)
Asian	10 (12)	16 (39)	17 (29)	13 (10)
African Am.	17 (21)	14 (34)	13 (22)	16 (12)
Other/Pac.Isl.	6 (7)	2 (6)	3 (5)	1 (1)

Medical group–percent (number)				

Cardiology	24 (29)	28 (70)	28 (49)	28 (21)
Hospitalists	60 (73)	57 (141)	53 (92)	65 (49)
Internist	2 (2)	4 (9)	4 (7)	3 (2)
Orthopedics	13 (16)	9 (23)	12 (20)	4 (3)
Gynecology	0 (0)	1 (2)	1 (2)	0 (0)
Cardiothoracic surgery	1 (1)	1 (2)	1 (2)	0 (0)

Overall health–percent (number)				

Excellent	5 (6)	6 (14)	5 (8)	8 (6)
Very good	20 (23)	20 (46)	20 (32)	20 (14)
Good	28 (32)	32 (73)	29 (46)	38 (27)
Fair	35 (39)	28 (65)	30 (48)	24 (17)
Poor	12 (13)	14 (31)	15 (24)	10 (7)

Heath scores at admission–mean (SD)				

Overall health (avg.)	3.3 (1.1)	3.2 (1.1)	3.3 (1.1)	3.1 (1.1)
HADS-anxiety**	7.7 (4.5)	7.1 (4.5)	7.9 (4.5)	5.3 (3.7)
HADS-depression***	5.6 (3.9)	5.4 (3.9)	5.8 (4.1)	4.5 (3.4)

HADS: hospital anxiety and depression scale.

*Maximum number of data points available for each group for this analysis. Individual outcome variables may have fewer data points available due to additional missing data.

**P < .001 between those accepting and refusing acupuncture—more anxious tend to accept acupuncture.

***P = .017 between those accepting and refusing acupuncture—more depressed tend to accept acupuncture.

**Table 2:** Comparison of study group numbers and acceptance of acupuncture when offered by all-patient refined diagnosis-related group (APR-DRG).

Diagnosis grouping (APR-DRG codes)	Not offered acupuncture percent (#)	Offered acupuncture percent (#)	Proportion who accepted when offered acupuncture
TOTAL	100 (117)	100 (228)	0.69
Stroke (45–47)	3 (3)	2 (5)	0.80
Pulmonary (121–143)	5 (6)	8 (19)	0.89
Pneumonia (139)	3 (3)	4 (8)	0.88
COPD (140)	0 (0)	3 (7)	1.00
Cardiovascular (161–207)	32 (38)	33 (75)	0.65
Bypass (165–166)	3 (3)	4 (9)	0.78
PCI with AMI (174)	9 (11)	9 (21)	0.76
Heart failure (194)	8 (9)	4 (8)	0.50
Gastrointestinal (221–284)	11 (13)	9 (20)	0.75
Musculoskeletal (300–347)	37 (43)	36 (81)	0.69
Hip/knee (302)	24 (28)	20 (45)	0.62
Total knee (8154*)	16 (19)	11 (26)	0.58
Total hip (8151*)	4 (5)	7 (16)	0.56
Back fusion (304)	2 (2)	4 (9)	0.89
Intervertebral disc (310)	5 (6)	7 (15)	0.87
Uterine procedures (513)	5 (6)	4 (8)	0.50
Other	7 (8)	9 (20)	0.65

*Procedure codes.

COPD: chronic obstructive pulmonary disease; PCI: percutaneous coronary interventions; AMI: acute myocardial infarction.

**Table 3:** Average ratio of actual-to-expected LOS and total costs by APR-DRG group for groups with *N* > 10.

		Not offered		Offered		*P* value	
Diagnosis grouping (APR-DRGs)	N*	LOS mean (SD)	Total costs mean (SD)	LOS mean (SD)	Total costs mean (SD)	LOS	costs
All participants	336	0.94 (0.4)	0.95 (0.5)	1.04 (0.5)	1.00 (0.5)	.108	.308
Pulmonary (121–143)	25	0.90 (0.7)	0.77 (0.5)	1.16 (0.6)	1.30 (0.8)	.123	.391
Pneumonia (139)	11	1.41 (0.7)	1.11 (0.6)	1.21 (0.7)	1.51 (0.9)	.702	.504
Cardiovascular (161–207)	111	1.00 (0.5)	0.95 (0.3)	1.16 (0.6)	1.07 (0.4)	.170	.147
Bypass (165–166)	12	1.09 (0.3)	0.85 (0.1)	0.93 (0.2)	0.95 (0.2)	.341	.512
PCI w/AMI (174)	32	1.06 (0.4)	1.00 (0.2)	1.04 (0.5)	1.01 (0.3)	.876	.927
Heart failure (194)	17	0.84 (0.3)	0.80 (0.2)	1.38 (0.6)	1.49 (0.7)	.049	.021
Gastrointestinal (221–284)	32	0.79 (0.3)	0.91 (0.6)	0.98 (0.5)	0.89 (0.4)	.266	.901
Musculoskeletal (300–347)	120	0.94 (0.4)	0.89 (0.2)	0.93 (0.4)	0.91 (0.3)	.868	.773
Hip/knee (302)	73	1.01 (0.3)	0.96 (0.2)	0.97 (0.4)	1.00 (0.2)	.636	.439
Total knee (8154^†^)	45	1.01 (0.4)	0.98 (0.2)	1.01 (0.5)	0.95 (0.2)	.993	.612
Total hip (8151^†^)	21	1.03 (0.3)	1.00 (0.1)	0.96 (0.3)	1.10 (0.2)	.659	.350
Back fusion (304)	11	0.81 (0.2)	0.76 (0.2)	0.75 (0.2)	0.72 (0.2)	.476	.476
Intervertebral disc (310)	21	0.67 (0.1)	0.70 (0.1)	0.96 (0.5)	0.79 (0.3)	.040	.362
Uterine procedures (513)	14	0.82 (0.2)	0.85 (0.1)	0.98 (0.2)	0.95 (0.2)	.373	.373

*N may differ from that shown in Table 2 since estimates of expected LOS and costs were not available for all participants.

COPD: chronic obstructive pulmonary disease; PCI: percutaneous coronary interventions; AMI: acute myocardial infarction.
